# Supplementary material for: HIVprotI: an integrated web based platform for prediction and design of HIV proteins inhibitors
Source: J Cheminform. 2018 Mar 9;10:12. doi: 10.1186/s13321-018-0266-y (PMC5845081; doi:10.1186/s13321-018-0266-y)
Supplement: Supplementary file 2 — Additional file 2. Source code of HIVProtI web server. [file 13321_2018_266_MOESM2_ESM.zip › HIVprotI_Source-code/hivprotI/weka-3-6-6/doc/weka/classifiers/lazy/kstar/KStarCache.TableEntry.html]

KStarCache.TableEntry


---


|  |  |  |
| --- | --- | --- |
| **Overview** \| **Package** \| **Class** \| **Tree** \| **Deprecated** \| **Index** \| **Help** \| **Weka's home** | |  |
| **PREV CLASS**   **NEXT CLASS** | **FRAMES**    **NO FRAMES**     **All Classes** |  |
| SUMMARY: NESTED \| FIELD \| CONSTR \| METHOD | DETAIL: FIELD \| CONSTR \| METHOD |  |


---


## weka.classifiers.lazy.kstar Class KStarCache.TableEntry

```
java.lang.Object
  weka.classifiers.lazy.kstar.KStarCache.TableEntry
```

**All Implemented Interfaces:**: java.io.Serializable, RevisionHandler

**Enclosing class:**: KStarCache

---

``` public class KStarCache.TableEntry extends java.lang.Object implements java.io.Serializable, RevisionHandler ```

Hashtable collision list.

**See Also:**: Serialized Form

---

| **Field Summary** | |
| --- | --- |
| `int` | `hash`             attribute value hash code |
| `double` | `key`             attribute value |
| `KStarCache.TableEntry` | `next`             next table entry (separate chaining) |
| `double` | `pmiss`             transformation probability to missing value |
| `double` | `value`             scale factor or stop parameter |


**Constructor Summary**

`KStarCache.TableEntry(int hash,
double key,
double value,
double pmiss,
KStarCache.TableEntry next)`
  
          Constructor


| **Method Summary** | |
| --- | --- |
| `java.lang.String` | `getRevision()`             Returns the revision string. |

**Methods inherited from class java.lang.Object**

`equals, getClass, hashCode, notify, notifyAll, toString, wait, wait, wait`

**Field Detail**

### hash

```
public int hash
```

:   attribute value hash code

---


### key

```
public double key
```

:   attribute value

---


### value

```
public double value
```

:   scale factor or stop parameter

---


### pmiss

```
public double pmiss
```

:   transformation probability to missing value

---


### next

```
public KStarCache.TableEntry next
```

:   next table entry (separate chaining)


**Constructor Detail**

### KStarCache.TableEntry

```
public KStarCache.TableEntry(int hash,
                             double key,
                             double value,
                             double pmiss,
                             KStarCache.TableEntry next)
```

:   Constructor


**Method Detail**

### getRevision

```
public java.lang.String getRevision()
```

:   Returns the revision string.

    :   **Specified by:**: `getRevision` in interface `RevisionHandler`
    :   **Returns:**: the revision


---


|  |  |  |
| --- | --- | --- |
| **Overview** \| **Package** \| **Class** \| **Tree** \| **Deprecated** \| **Index** \| **Help** \| **Weka's home** | |  |
| **PREV CLASS**   **NEXT CLASS** | **FRAMES**    **NO FRAMES**     **All Classes** |  |
| SUMMARY: NESTED \| FIELD \| CONSTR \| METHOD | DETAIL: FIELD \| CONSTR \| METHOD |  |


---
